# Denosumab discontinuation: COVID-19 pandemic and beyond

**DOI:** 10.1093/jbmrpl/ziae046

**Published:** 2024-04-02

**Authors:** Salvatore Minisola

**Affiliations:** Department of Clinical, Internal, Anaesthesiologic and Cardiovascular Sciences, “Sapienza” University of Rome

**Keywords:** denosumab, osteoporosis, COVID-19, fracture, adherence

## Abstract

The coronavirus disease 2019 (COVID-19) pandemic triggered significant disruptions in health care systems around the world, with a particularly heavy impact on patients with chronic diseases. A number of studies have shown an immediate decrease in on-time denosumab therapy at the start of COVID-19 pandemic. However, independent of the “emergency” that occurred during the COVID-19 pandemic, there are other situations in which denosumab is discontinued. In such situations, it is important to have a programmed strategy to optimize care while limiting the risk for unwanted outcomes.

The coronavirus disease 2019 (COVID-19) pandemic triggered significant disruptions in health care systems around the world, with a particularly heavy impact on patients with chronic diseases. Indeed, most patients experienced a gap in care for reasons including unavailability of healthcare professionals, lockdown measures, and medical activity interruptions. This gap was most pronounced for therapies requiring specific healthcare requirements for administration, as in the case of parenteral therapies.^1^

In this context, Rzepka and coworkers report the effect of the COVID-19 pandemic on adherence to denosumab therapy.^2^ They show an immediate decrease in on-time denosumab therapy at the start of the COVID-19 pandemic, in line with previous studies that also demonstrate that discontinuation of denosumab therapy was also complicated by an increase in vertebral fracture occurrence.^3^^,^^4^ Rzepka *et al.* also show that the COVID-19 pandemic had a minimal effect on adherence in patients residing in nursing home when compared with ambulatory patients. This finding highlights how the inability to reach external medical resources was the most important factor driving lower adherence.^2^

Interruption of denosumab therapy without taking necessary countermeasures to limit the surge in osteoclast activity following denosumab discontinuation may be followed by catastrophic effects, especially in those taking the drug for a long time. This “rebound phenomenon” following denosumab discontinuation is a well-described clinical condition since its initial description.^5^ From a biochemical point of view, this is characterized by an increase in markers of both bone formation and resorption. This leads to a negative skeletal balance with a rapid decrease of bone mineral density in addition to the most feared consequence—an increased incidence of vertebral fractures. A short off-treatment period of 2–10 months is enough to produce these deleterious consequences. The pathological mechanism underlying this sequence of events is related to the process of osteoclast recycling. Inhibition of nuclear factor kappa-B ligand (RANKL) by denosumab leads to the fission of osteoclasts into osteomorphs which then recycle to form resorbing osteoclasts once RANKL inhibition is withdrawn.^6^ Therefore, once denosumab is discontinued, there is an accrual of osteoclast precursors and osteomorphs which are primed to immediately differentiate into osteoclasts and resorb bone. This sequence of events provides rationale for the fact that negative skeletal consequences are worst in those with longer duration of denosumab treatment.

Independent of the “emergency” during the COVID-19 pandemic, other situations occur in which denosumab is discontinued (Table 1). In such situations, it is important to have a programmed strategy to optimize care while limiting the risk for unwanted outcomes. Indeed, a number of studies have been published exploring the best therapeutic option to overcome the osteoclast rebound and its associated increased incidence of vertebral fractures. It is now considered mandatory to initiate another antiresorptive treatment if denosumab therapy is stopped. Both zoledronic acid and alendronate seem to mitigate bone mineral density loss, while lesser effects are seen with other antiresorptive agents.^7^^,^^8^ Since local production of osteoprotegerin by mature osteoblasts play a critical role in suppressing RANKL activity and osteoclastogenesis, romosozumab (a monoclonal antibody against sclerostin) could have a role. Accordingly, recently Ebina and coworkers showed that administration of romosozumab after denosumab was not followed by a decrease in bone mineral density at either the lumbar or hip sites despite significant increases in N-terminal type I procollagen propeptide levels and tartrate resistant acid phosphatase isoform 5b activity.^9^ It remains unclear at this time if these changes have a neutral effect on fracture incidence following transition from denosumab to romosozumab. Finally, Kahn and coworkers recently observed no bone mineral density loss after patients were switched from the 60 mg dose of denosumab to a 30-mg dose for as long as the treatment was continued. They hypothesized that that this approach could be a strategic option for weaning from such therapy.^10^ However, both such approaches are best considered if there is an “up front” strategy in place at the time of inception of denosumab therapy.

**Table 1:** Conditions associated with denosumab discontinuation.

1. Inability to reach health services (in case of COVID-19, other pandemics or natural disasters)
2. Patient refusal for further treatment
3. Dental care (ie, invasive dental procedures) with denosumab discontinuation without notification of the prescribing physician
4. Side effects (ie, osteonecrosis of the jaw)
5. Change in therapy due to inefficacy and incident fractures
6. BMD goal achievement (no previous fracture and no longer at high risk of fracture)
7. Discontinuation of adjuvant therapy (no previous fracture and no longer at high risk of fracture) when cancer treatment is completed
8. Insurance coverage issues

The use of telemedicine to increase patient awareness regarding issues associated with denosumab discontinuation and other approaches can be considered when determining how best to maintain patients on denosumab treatment. Alternatively, the involvement of nurses or community pharmacists for drug delivery at home in countries where denosumab must be administered by a healthcare professional may represent a viable option. However, Rzepka and coworkers reported on denosumab discontinuation in an acute and unforeseen situation in which different measures, including those not previously considered, should be adopted. Strategies to improve the timely administration of denosumab in unusual clinical settings such as pandemics and natural, public health, environmental, or nuclear disasters are needed. Options including the provision of reminders from health systems delivered by auto-dialers, patient portals, or text messaging may be other reasonable options.

In conclusion, Rzepka and coworkers highlight a clinical situation often faced by physicians, that of untimely denosumab discontinuation. This dilemma has unfortunately become more frequently encountered given the very large number of patients who have already received 10 or more years of denosumab therapy given the current absence of any trial of denosumab treatment with a time interval that exceeds this current upper limit.

## Author contributions

Salvatore Minisola (Conceptualization, Writing—review & editing)

## Funding

None declared.

## Conflicts of interest

S.M. served as a speaker for Abiogen, Beijing Society Biomedical Engineering, Bruno Farmaceutici, Diasorin, Geopharma, Sandoz, UCB. He also served in advisory board of Abiogen, Ely Lilli, Kyowa Kirin, Faes Farma., Novo Nordisk, UCB.
